# EpCAM peptide-primed dendritic cell vaccination confers significant anti-tumor immunity in hepatocellular carcinoma cells

**DOI:** 10.1371/journal.pone.0190638

**Published:** 2018-01-03

**Authors:** Yoo Jin Choi, Seong-Joon Park, You-Soo Park, Hee Sung Park, Kwang Mo Yang, Kyu Heo

**Affiliations:** 1 Research Center, Dongnam Institute of Radiological & Medical Sciences, Busan, Republic of Korea; 2 Department of Radiation Oncology, Dongnam Institute of Radiological & Medical Sciences, Busan, Republic of Korea; 3 Department of Radiation Oncology, Korea Institute of Radiological & Medical Sciences, Seoul, Republic of Korea; Southern Illinois University School of Medicine, UNITED STATES

## Abstract

Cancer stem-like cells (CSCs) may play a key role in tumor initiation, self-renewal, differentiation, and resistance to current treatments. Dendritic cells (DCs) play a vital role in host immune reactions as well as antigen presentation. In this study, we explored the suitability of using CSC peptides as antigen sources for DC vaccination against human breast cancer and hepatocellular carcinoma (HCC) with the aim of achieving CSC targeting and enhancing anti-tumor immunity. CD44 is used as a CSC marker for breast cancer and EpCAM is used as a CSC marker for HCC. We selected CD44 and EpCAM peptides that bind to HLA-A2 molecules on the basis of their binding affinity, as determined by a peptide-T2 binding assay. Our data showed that CSCs express high levels of tumor-associated antigens (TAAs) as well as major histocompatibility complex (MHC) molecules. Pulsing DCs with CD44 and EpCAM peptides resulted in the efficient generation of mature DCs (mDCs), thus enhancing T cell stimulation and generating potent cytotoxic T lymphocytes (CTLs). The activation of CSC peptide-specific immune responses by the DC vaccine in combination with standard chemotherapy may provide better clinical outcomes in advanced carcinomas.

## Introduction

Tumor cells express antigens that can be recognized by the immune system of their host. Cancer patients can be inoculated by these tumor-associated antigens (TAAs) to induce systemic immune responses that may result in the destruction of various cancers. This procedure is defined as active immunotherapy, or vaccination [1].

Dendritic cells (DCs) are the most potent professional antigen-presenting cells (APCs) that exist in the immune system [2, 3]. DC vaccines aim to stimulate cancer-specific effector T cells to eradicate tumor cells and to stimulate immunological memory to control cancer recurrence [4]. Human DCs are commonly generated from monocytes that are isolated from peripheral blood mononuclear cells (PBMCs) and differentiated to produce immature DCs (iDCs). The iDCs then undergo maturation and an antigen-loading step to produce mature DCs (mDCs) [5]. DCs have been pulsed/activated with tumor lysates, recombinant proteins, or peptides, and peptide pulsing has been most widely investigated [6–10]. Studies have shown that peptide-pulsed DCs can present antigens to naïve T lymphocytes, and in turn activate and induce T lymphocytes to become antigen-specific cytotoxic T lymphocytes (CTLs) that target tumor cells [11]. Both the proliferative and cytolytic functions of tumor-specific CTLs require antigen recognition by the T cell receptor (TCR) in the context of major histocompatibility complex class one (MHC class I) molecules presented on APCs or target cells [12].

Hepatocellular carcinoma (HCC) is a malignant disease that is often associated with a very poor prognosis [13]. While considerable efforts have been made to improve HCC treatment—which mainly depends on surgical resection, liver transplantation and chemotherapy—the HCC mortality rate remains high, largely due to cancer recurrence after surgery or intra-hepatic metastasis that develop through invasion of the portal vein or spread to other parts of the liver [14]. Breast cancer ranks first among the causes of mortality among females aged between 20 and 59 years [15]. In recent years, the encouraging trend towards earlier detection and the increased use of systemic adjuvant treatments have improved breast cancer survival rates; however, nearly half of all breast cancer patients treated for localized disease develop metastasis [16]. Cancer stem-like cells (CSCs) typically represent a small fraction of tumor cells that can self-renew and differentiate into many more mature cancer cells [17]. The failure of conventional cancer therapy may be due to the presence of residual CSCs that can survive in a dormant state for many years after remission and result in tumor relapse [18].

In the present study, we investigated the effect of CSC peptides as antigen sources for DC vaccination against human breast cancer and HCC. Our results revealed that pulsing DCs with CD44 or EpCAM peptides enhanced T cell stimulation thus resulting in the induction of cell cytotoxicity. Furthermore, pulsing DCs with EpCAM peptides significantly suppressed tumor growth. The results of the present study suggest that the capacity of this vaccine to target CSCs could be exploited as a novel therapeutic strategy to inhibit tumor relapse.

## Materials and methods

### Cell culture conditions

The human breast adenocarcinoma cell line MCF-7 and the human hepatoma cell line HepG2 were purchased from the American Type Culture Collection (Rockville, MD, USA) and cultured in DMEM (Welgene, Daegu, Korea) supplemented with 10% fetal bovine serum (FBS) (HyClone, Logan, UT, USA) and 100 U/ml penicillin/streptomycin (Gibco, Carlsbad, CA, USA) in a humidified atmosphere with 5% CO_2_ at 37°C.

### Flow cytometry and cell sorting

Cells were trypsinized and suspended in phosphate-buffered saline (PBS) containing 2% FBS at a density of 1×10^8^ cells/ml. For flow cytometry, the MCF-7 cells were incubated with anti-CD24-FITC and anti-CD44-APC monoclonal antibodies (mAbs) (BD Biosciences, Bedford, MA, USA), and the HepG2 cells were incubated with the anti-EpCAM-PerCP-Cy5.5 mAb (BD Biosciences, San Jose, CA, USA) on ice for 60 min. FITC mouse anti-IgG2a, APC mouse anti-IgG2b and PerCP/Cy5.5 anti-mouse IgG1 (BD Biosciences) were used as isotype control antibodies. After being washed with PBS supplemented with 1% FBS, the labeled cells were sorted on a FACSAria Cell Sorter (BD Biosciences, San Jose, CA, USA).

### Cell lysate preparation

Cell pellets were resuspended in an equal volume of Cellgro (Genix, Freiburg, Germany), and the suspensions were sonicated on ice over 4-min intervals, which included pulsing for 15 seconds on and 15 seconds off, and they were then centrifuged at 14,000 rpm for 30 min. The supernatants were filtered with a 0.2-μm syringe filter, and the protein concentrations were determined using Bradford protein assay reagent (Bio-Rad, Hercules, CA, USA). Supernatants were stored at -80°C until use.

### Western blot analysis

Protein samples were separated by 10% SDS–PAGE and transferred to polyvinylidene difluoride membranes. The membranes were incubated with anti-CD44 (1:200; sc-53298; Santa Cruz Biotechnology, Dallas, TX, USA), anti-ALDH1/2 (1:200; sc-50385; Santa Cruz Biotechnology), anti-EpCAM (1:200; sc-25308; Santa Cruz Biotechnology) or anti-GAPDH (1:2000; sc-32233; Santa Cruz Biotechnology). Antibody immunostaining was achieved using the Super-Signal West Pico enhanced chemiluminescence substrate, and the proteins were detected using the LAS-3000 PLUS image analyzer (Fuji Photo Film Co., Kanagawa, Japan).

### T2 cell binding assay

The binding affinities of all 9-mer peptides were predicted for HLA class I alleles. Binding prediction analysis was performed using a computer algorithm from the Immune Epitope Database (IEDB) website, and eight peptides with an amino acid sequence motif appropriate for binding to HLA-A2 were identified. The peptides were synthesized by AbFrontier (Seoul, Korea). The ability of the peptides to bind HLA-A2 molecules was measured using the T2 cell line because HLA-A2 expression is stabilized and maintained on the surface of T2 cells when peptides are bound in the grooves of HLA-A2 molecules to form complexes. T2 cells (2×10^6^/ml/well of a 24-well plate) were incubated in either medium alone or medium containing individual peptides at a concentration of 10 μg/ml plus 5 μg/ml β_2_-microglobulin (Sigma-Aldrich, St. Louis, Mo, USA) overnight. The HLA-A2 strong-binding HIV peptide ILKEPVHGV was used as a positive control in the peptide binding assays. After being incubated with medium or medium plus peptide, the T2 cells were washed with PBS and incubated with an anti-HLA-A2-PE mAb (BD Biosciences, BB7.2 clone) for 1 h at 4°C. The fluorescence intensity and positive cell percentages were measured on a flow cytometer (BD FACSAria, San Jose, CA, USA). The ability of each peptide to bind HLA-A2 was evaluated by determining the mean fluorescence intensity of stained T2 cells that were pulsed with the peptides.

### iDC generation

Human DCs were generated from monocytes isolated from the PBMCs of healthy HLA-A2 donors using Histopaque^®^-1077 (Sigma-Aldrich). The PBMCs were resuspended in x-Vivo medium (Lonza, Walkersville, MD, USA) containing 1% human serum (Sigma-Aldrich) at a cell density of 1×10^7^ cells/ml and plated in T75 flasks. The flasks were incubated in 5% CO_2_ at 37°C for 90 min, and nonadherent cells were then gently resuspended and removed. The adherent cells were then cultured in LGM-3 medium (Lonza, Walkersville, MD, USA) containing 1% human serum, recombinant human GM-CSF (1,000 units/ml, R&D Systems, USA) and IL-4 (1,000 units/ml, R&D Systems, USA) for 5 days. Next, the adherent cells were harvested, counted, and resuspended in culture medium. The phenotypes of iDCs were determined by flow cytometry using PE-conjugated anti-CD83 (1:20; IM2218U; Beckman Coulter, Marseille, France), anti-HLA-ABC (1:20; IM1838U, Beckman Coulter) and PE-conjugated anti-HLA-DR (1:20; IM1639, Beckman Coulter) antibodies. The expression of cell surface markers was then determined by flow cytometry using the Cytomics FC 500 instrument (Beckman Coulter, Fullerton, CA, USA).

### DC antigen loading

After DC generation, 5×10^6^ iDCs were stimulated with tumor cell lysates and peptides (500 μg/ml) in the presence of GM-CSF and IL-4. After 4 h, the maturation cocktail [1 μg/ml TNF-α, IL-1β, IL-6 (all from R&D Systems), and 10 μg/ml prostaglandin E2 (PGE2) (Sigma-Aldrich)] was added, and the cells were incubated with the cocktail for 2 days.

### Autologous T cell activation by DCs sensitized with target cells

PBMCs were incubated in 5% CO_2_ at 37°C for 90 min, and nonadherent cells were then gently resuspended and harvested. DCs were transferred to new 6-well plates and cultured with T cells in LGM-3 medium containing 1% human serum, GM-CSF and IL-4 for 5 days. Fresh medium containing cytokines was added each day.

### T cell cytotoxic activity assay

T cell cytotoxicity is presented as the percentage of target cells that were lysed. The target cells were labeled with 5 μM Carboxifluorescein diacetate N-succinimidyl ester (CSFE) (eBioscience, USA). After labeling, the cells were washed and resuspended at 1×10^5^ cells/ml in LGM-3 medium (Lonza) supplemented with 1% human serum, GM-CSF, IL-4 and IL-2 (100 units/ml). The T cells were incubated with 1×10^4^ target cells in each FACS tube at various culture ratios (T cell: target cell = 10:1, 20:1 and 40:1), and the FACS tubes were incubated at 37°C for 6 h in a humidified, 5% CO_2_ incubator. After incubation, the cells were stained with 50 μg/ml propidium iodide (PI) (Sigma-Aldrich), a red dye that penetrates the membranes of dying cells, prior to analysis by flow cytometry (Beckman Coulter FC500, USA). The cytotoxicity was calculated as the percentage of PI-positive cells among the CFSE-positive target cells.

### Enzyme-linked immunosorbent assay (ELISA)

The IL-12 and IL-7 that were released by mDCs were detected using the Human IL-12p70 ELISA Kit (Neobioscience Technology, China) and the Human IL-7 ELISA Kit (RapidBio, California, USA), respectively, according to the manufacturer’s protocols. IFN-γ secretion by CTLs was detected using the Human IFN-γ ELISA Kit (BD Biosciences, USA) according to the manufacturer’s protocol. The optical density (OD) of samples was assessed at 550 nm using a microtiter plate spectrophotometer (Beckman Coulter detection platform, USA).

### Animal studies

Female BALB/c nude mice (4 weeks old) were purchased from the Central Lab at Animal Inc. (Seoul, Korea). All animal procedures were performed according to the protocol approved by the Dongnam Institute of Radiological and Medical Sciences Institutional Animal Care and Use Committee. HepG2 cells in the logarithmic growth phase (2×10^6^ cells/50 μl saline) were subcutaneously inoculated into the right flanks of 5-week-old mice. When the tumors grew to a size of approximately 35 mm^3^ (after approximately 10 days), the mice were stratified into groups of 5 animals that had equal mean tumor volumes and were intravenously (i.v.) treated with CTLs (EpCAM peptide-stimulated DC-induced CTLs, HepG2 total lysate-stimulated DC-induced CTLs, DC-induced CTLs or unstimulated T cells) on days 10 and 17 after tumor implantation. The tumor sizes were measured weekly using calipers, and the tumor volumes were calculated using the following formula: (width)^2^ × length × 0.52. After 7 days, the mice were sacrificed using abdominal arterial blood collection method to obtain the lymphocytes from the spleen.

### Immunohistochemistry

Paraffin-embedded sections were prepared from tumor tissue samples that had been previously fixed in 4% buffered formalin. Immunohistochemistry was performed on the deparaffinized sections that had been pretreated for antigen retrieval with EDTA Tris-HCl buffer [distilled water (DW): 1 M Tris-HCl (pH 9.0): 0.25 M EDTA (pH 7.0) = 500:5:2] in a microwave for 15 min. Endogenous peroxidase activity was quenched with 0.3% H_2_O_2_ for 10 min in DW. To reduce background staining, the sections were treated for 1 h with 5% normal horse serum (VECTASTAIN ABC HRP Kit). The samples were incubated with anti-EpCAM (Santa Cruz biotechnology) in a humidified chamber at 4°C overnight. Next, the samples were incubated for 1 h with secondary mAbs, a biotinylated antibody and the ABC complex (VECTASTAIN ABC HRP Kit). The samples were dyed with a DAB Peroxidase Substrate Kit (Vector Lab, USA). The cells were counterstained using hematoxylin (Sigma-Aldrich, USA), and the immunohistochemistry images were collected using an upright microscope (Nikon ECLIPSE 80i, Japan).

### Statistical analysis

The results are expressed as the means ± standard deviation (SD). Significant differences between the treatments and control were evaluated by ANOVA (followed by the Dunnett’s test). A *p-*value less than 0.05 was defined as significant.

## Results

### Identification of HLA-A2-restricted peptides

CSCs are a subset of cancer cells bearing stem cell features such as self-renewal and the ability to differentiate into progeny cells, and play a pivotal role in cancer initiation, progression and recurrence [17]. Several cell surface markers for separating CSCs have been identified in various types of cancers. CD44^+^CD24^-^ subpopulation has been regarded as a marker of CSCs in breast cancer cells. In HCC, EpCAM^+^ cells have been reported to have CSC properties [19].

We screened partial amino acid sequences of CD44 and EpCAM for HLA-A2-binding peptides using a peptide binding database. The IEDB is based on the predicted binding strength between a specific peptide and related MHC molecules. By comparing the predicted binding scores, we identified eight peptides for each marker (CD44 and EpCAM) that have high affinity for HLA-A2 molecules.

The predicted HLA-A2-binding affinities of the peptides were measured using the T2 cell binding assay. A peptide from HIV type 1 reverse transcriptase (HIV-pol) was used as a positive control [20]. As shown in Table 1, the strongest binding affinity for TAAs was observed with the HLA-A2 molecule. Good and intermediate binders were chosen for subsequent DC vaccination-based immunogenicity studies in MCF-7 and HepG2 cells.

**Table 1:** Binding affinity of TAA for HLA-A2 molecules.

Peptide sequence			Percentile rank ^a^	Mean fluorescent intensity ^b^
Media				17.2 ± 1.2
HIV-pol		ILKEPVHGV	2	66.6 ± 2.7 ^#^
CD44	MCF-7 lysates			34.3 ± 3.0 ^#^
	CD44+MCF7 lysates			40.9 ± 3.3 ^#^
	1	YIFYTFSTV	0.6	57.7 ± 2.1 ^#^ *
	2	LILAVCIAV	1	48.1 ± 2.9 ^#^ *
	3	SLLALALIL	1.7	56.0 ± 2.0 ^#^ *
	4	IILASLLAL	2.3	49.4 ± 2.4 ^#^ *
	5	WLIILASLL	3.4	50.3 ± 2.9 ^#^ *
	6	VLLQTTTRM	4.7	54.7 ± 1.6 ^#^ *
	7	GLVEDLDRT	6.9	44.0 ± 3.6 ^#^ *
	8	TVGDSNSNV	8.3	38.3 ± 4.2 ^#^
EpCAM	HepG2 lysates			32.9 ± 3.0 ^#^
	EpCAM+HepG2 lysates			38.2 ± 1.3 ^#^
	1	VVAGIVVLV	1.5	63.0 ± 2.0 ^#^ *
	2	GLKAGVIAV	2.0	51.8 ± 2.3 ^#^
	3	VLAFGLLLA	2.1	48.7 ± 2.8 ^#^
	4	RTYWIIIEL	2.1	52.5 ± 0.9 ^#^ *
	5	SMCWCVNTA	2.3	58.5 ± 2.8 ^#^ *
	6	SMQGLKAGV	2.3	47.9 ± 2.0 ^#^
	7	ILYENNVIT	3.8	56.0 ± 1.9 ^#^
	8	LLLAAATAT	4.8	35.2 ± 1.8 ^#^

^a^ CTL epitope prediction score as calculated by the IEDB software (http://www.iedb.org)

^b^ Mean fluorescent intensity of stained T2 cells pulsed with peptides.

The data are expressed as the mean±SD, and significant differences emerged between the treated groups with ANOVA followed by Dunnett’s test.

^#^ *P*<0.05, compared with Media.

* *P*<0.05, compared with the MCF-7 or HepG2 lysates.

### CD44 peptide-specific CTLs can effectively kill MCF-7 CSCs

**Fig 1:**
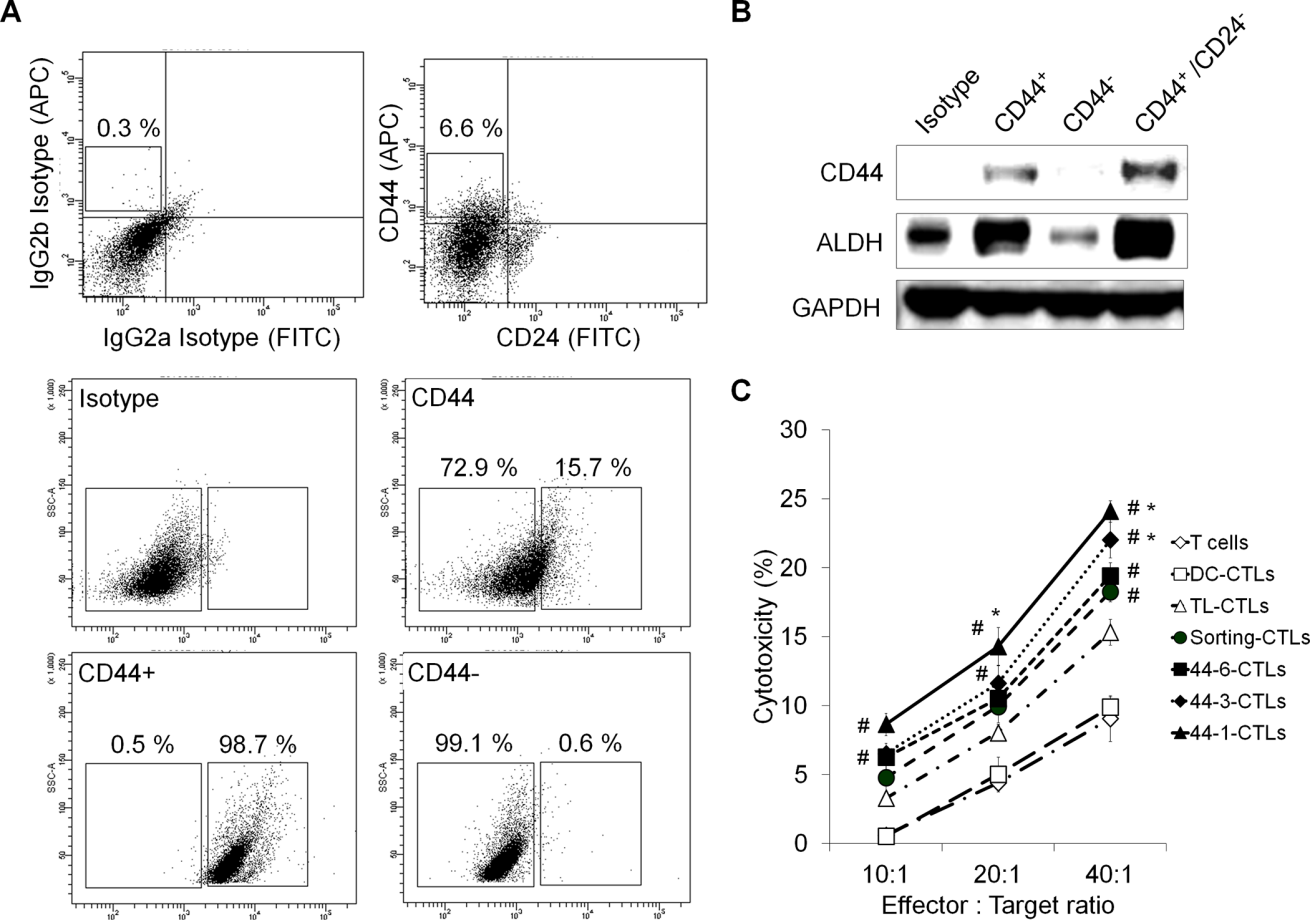
The specific lytic activity of MCF-7 cancer stem-like cell antigens. (A) Identification of populations of MCF-7 cells by flow cytometry analysis of CD44 and CD24 expression. (B) Western blot showing CD44 and ALDH protein expression in CD44^+^, CD44^-^ and CD44^+^CD24^-^ MCF7- cells. (C) Targeted killing of CD44^+^ MCF-7 cells by tumor-associated antigen-pulsed, DC-induced CTLs. The data are expressed as the mean±SD, and significant differences between the treated groups were detected using ANOVA followed by the Dunnett’s test. ^#^ *p*<0.05 compared with the DC-CTLs. * *p*<0.05 compared with the TL-CTLs.

A subpopulation (CD44^+^CD24^-^) of MCF-7 cells was separated and collected by flow cytometry (Fig 1A). To study whether CSCs from MCF-7 cells express certain TAAs, we measured the expression levels of aldehyde dehydrogenase (ALDH), a detoxifying metabolic enzyme that is often associated with stem and progenitor cell populations [21]. Western blot analysis was used to examine the expression of ALDH in the FACS-sorted cells. As shown in Fig 1B, ALDH was highly expressed in CD44^+^CD24^-^ cells and CD44^+^ cells.

DCs loaded with various antigens were co-cultured with T cells and CD44^+^ MCF-7 cells for 6 h. FACS analysis was performed to compare the cytotoxic activities of CTLs induced by unstimulated DCs (DC-CTLs), TL-CTLs (MCF-7 Tumor cell lysate stimulated DC-CTLs), Sorting-CTLs (CD44^+^ MCF-7 cell lysate stimulated DC-CTLs) and Pep-CTLs (which were induced by DCs stimulated with one of the following peptides: CD44-1, CD44-3 or CD44-6) against CD44^+^ MCF-7 cells. The results showed that Pep-CTLs had a more efficient cytotoxic activity against CD44^+^ MCF-7 cells than did Sorting-CTLs and TL-CTLs (Fig 1C).

Therefore, we hypothesized that CTLs stimulated by DCs pulsed with CD44 peptide can recognize the CD44 naturally present in breast cancer cells in the context of HLA-A2 and kill CD44 positive MCF-7 tumor cells.

### EpCAM^+^ HepG2 cells are more immunogenic

**Fig 2:**
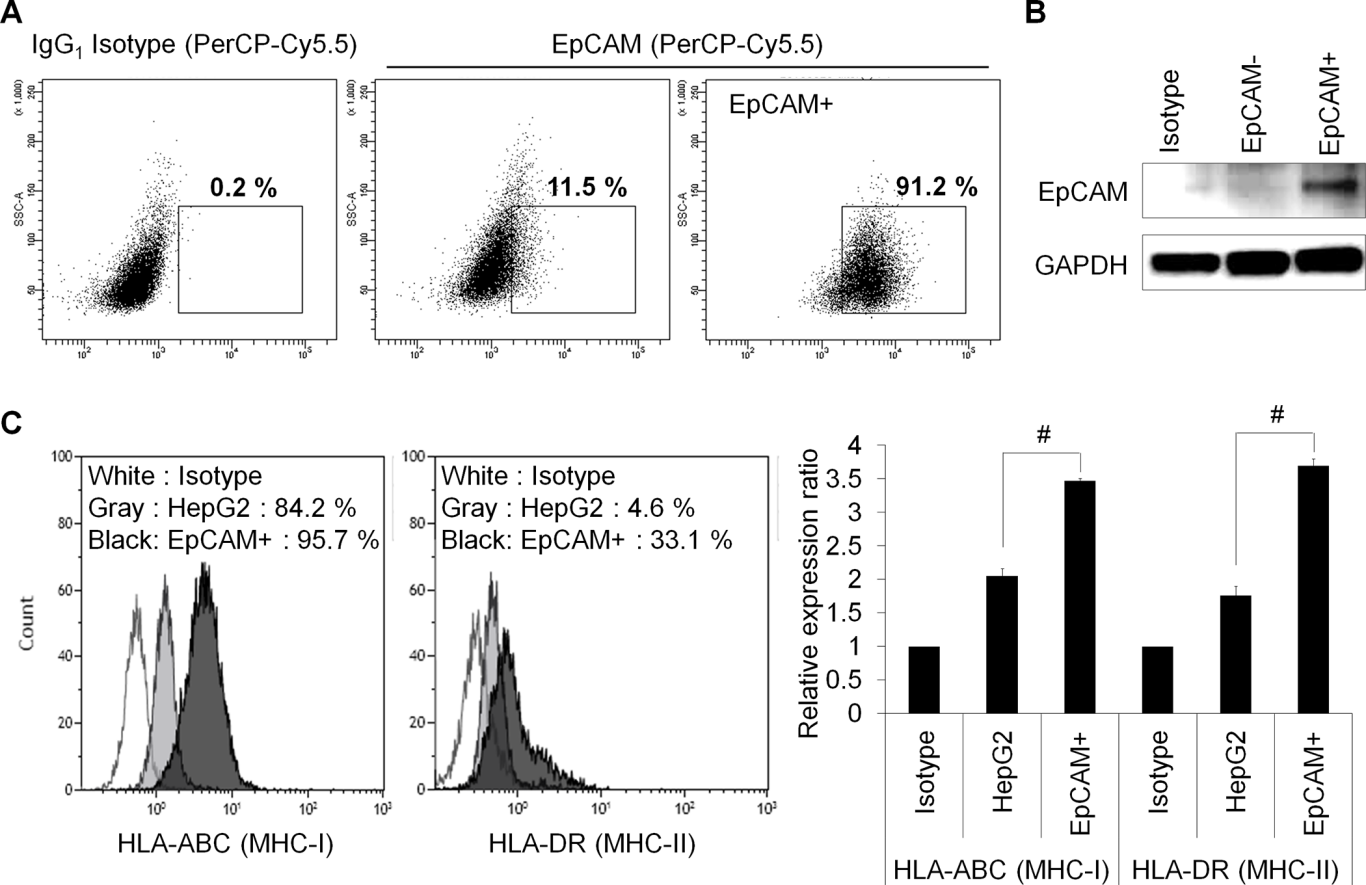
The immunological phenotype of HepG2 cancer stem-like cells. (A) The top 10% most brightly stained EpCAM positive HepG2 cells were purified by flow cytometry. A representative analysis of EpCAM^+^ HepG2 cell purity after sorting is shown. (B) The expression of EpCAM in EpCAM negative or positive HepG2 cells was evaluated using western blot analysis. (C) Flow cytometry analysis of HLA-ABC (MHC-I) and HLA-DR (MHC-II) expression in EpCAM^+^ HepG2 cells. HLA-ABC and HLA-DR expression in EpCAM^+^ HepG2 cells is indicated by the black areas. The data are expressed as the mean±SD, and significant differences between the treated groups were detected using ANOVA followed by the Dunnett’s test. ^#^ *p*<0.05 compared with the HepG2.

EpCAM^+^ HepG2 cells were sorted by flow cytometry, and after sorting, more than 90% of the purified cells expressed EpCAM (Fig 2A and 2B).

Tumor antigens must be presented by MHC class I molecules to be recognized by specific CTLs, and MHC class I molecule expression is essential for CTL activation [22]. Therefore, we evaluated the expression of MHC class I and class II molecules on CSCs. The expression of HLA-ABC (MHC-I) and HLA-DR (MHC-II) on EpCAM^+^ HepG2 cells was examined by flow cytometry. The MHC markers were expressed at higher levels on EpCAM^+^ HepG2 cells than on HepG2 cells, suggesting that EpCAM^+^ HepG2 cells are more immunogenic (Fig 2C).

To determine if CSC lysates and CSC peptides, which upregulate the HLA-A2 molecule, actually bind to the HLA-A2 molecule, we tested the binding affinity of EpCAM^+^ HepG2 cells and EpCAM-1 peptides using a T2 cell binding assay. As shown in Table 1, the EpCAM-1 peptide had a stronger binding affinity than that of EpCAM^+^ HepG2 cells for the HLA-A2 molecule.

### Characterization of DCs pulsed with HepG2 TAAs

**Fig 3:**
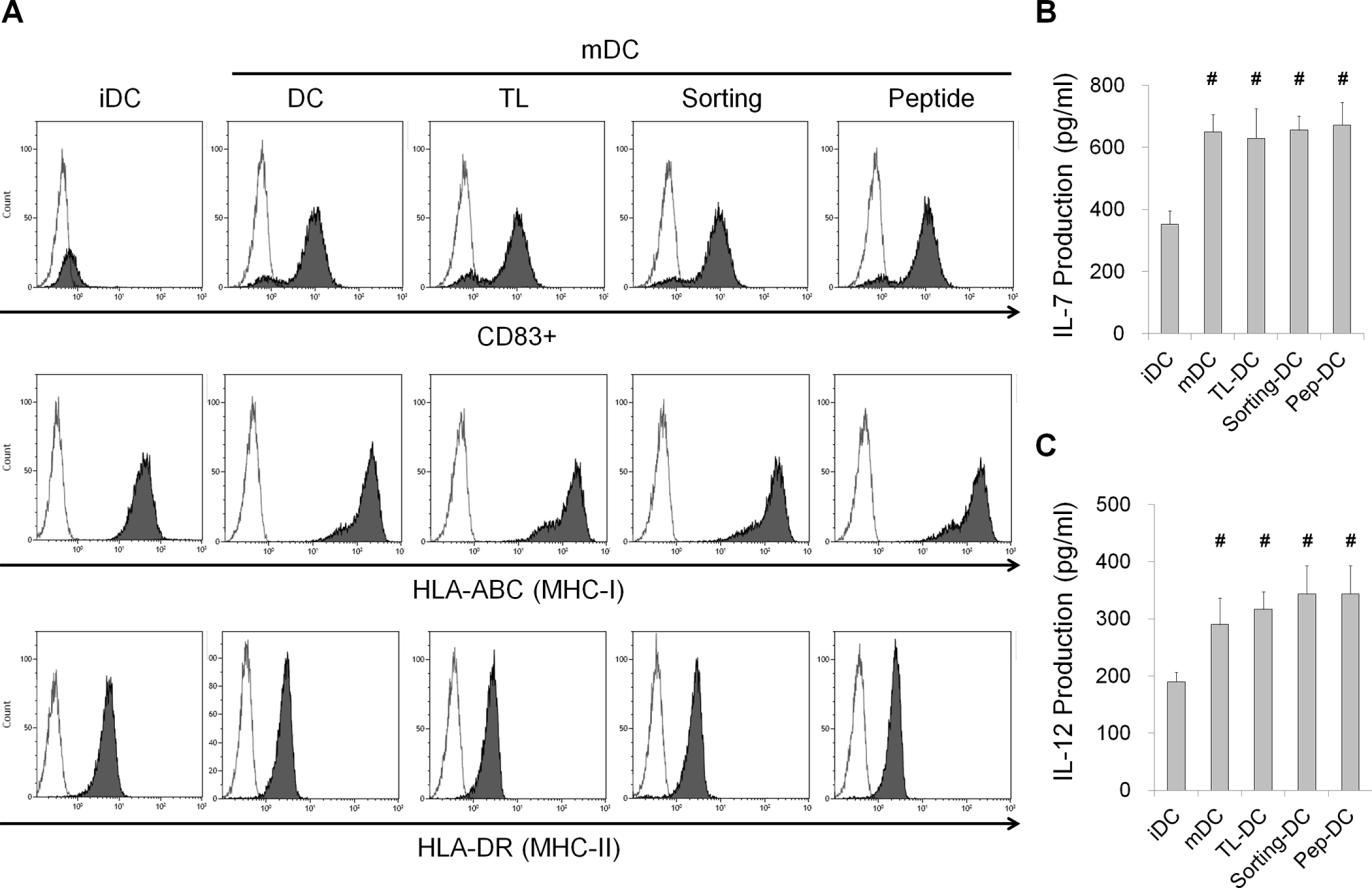
Analysis of DC surface markers and the cytokine secretion assay. (A) The immunophenotype of DCs pulsed with antigens. The mDC phenotypes were analyzed by flow cytometry using the indicated antibodies (black areas) and isotype controls (white areas). (B and C) IL-7 (B) and IL-12 (C) levels in DC culture supernatants, as determined by ELISA. The data are expressed as the mean±SD, and significant differences between the treated groups were detected using ANOVA followed by the Dunnett’s test. ^#^ *p*<0.05 compared with the iDCs.

As shown in Fig 3A, mDCs isolated from the PBMCs of a healthy HLA-A2^+^ donor were loaded with TL-DCs (HepG2 cell lysate stimulated), Sorting-DCs (EpCAM^+^ HepG2 cell lysate stimulated) or Pep-DCs (EpCAM-1 peptide stimulated). These human mDCs highly expressed CD83, HLA-ABC (MHC-I) and HLA-DR (MHC-II) at levels similar to those expressed by mDCs that were not loaded with antigens. Thus, the loading DCs with EpCAM peptides resulted in the efficient generation of mDCs without altering the phenotype of the mDCs.

Cytokine secretion is often assessed to determine DC function, and DCs reportedly produce IL-7 and IL-12, which contributes to T lymphocyte activation [23–24]. Therefore, we measured IL-7 and IL-12 production by iDCs and mDCs pulsed with or without TAAs. The concentrations of IL-7 and IL-12 in the mDC culture supernatants were significantly higher than those in the iDC culture supernatants. However, there were no significant differences in the concentrations of IL-7 and IL-12 among the TL-DCs, Sorting-DCs, Pep-DCs or mDCs pulsed without antigens in the culture supernatants (Fig 3B and 3C).

### EpCAM peptide-specific CTLs can effectively kill HepG2 CSCs

**Fig 4:**
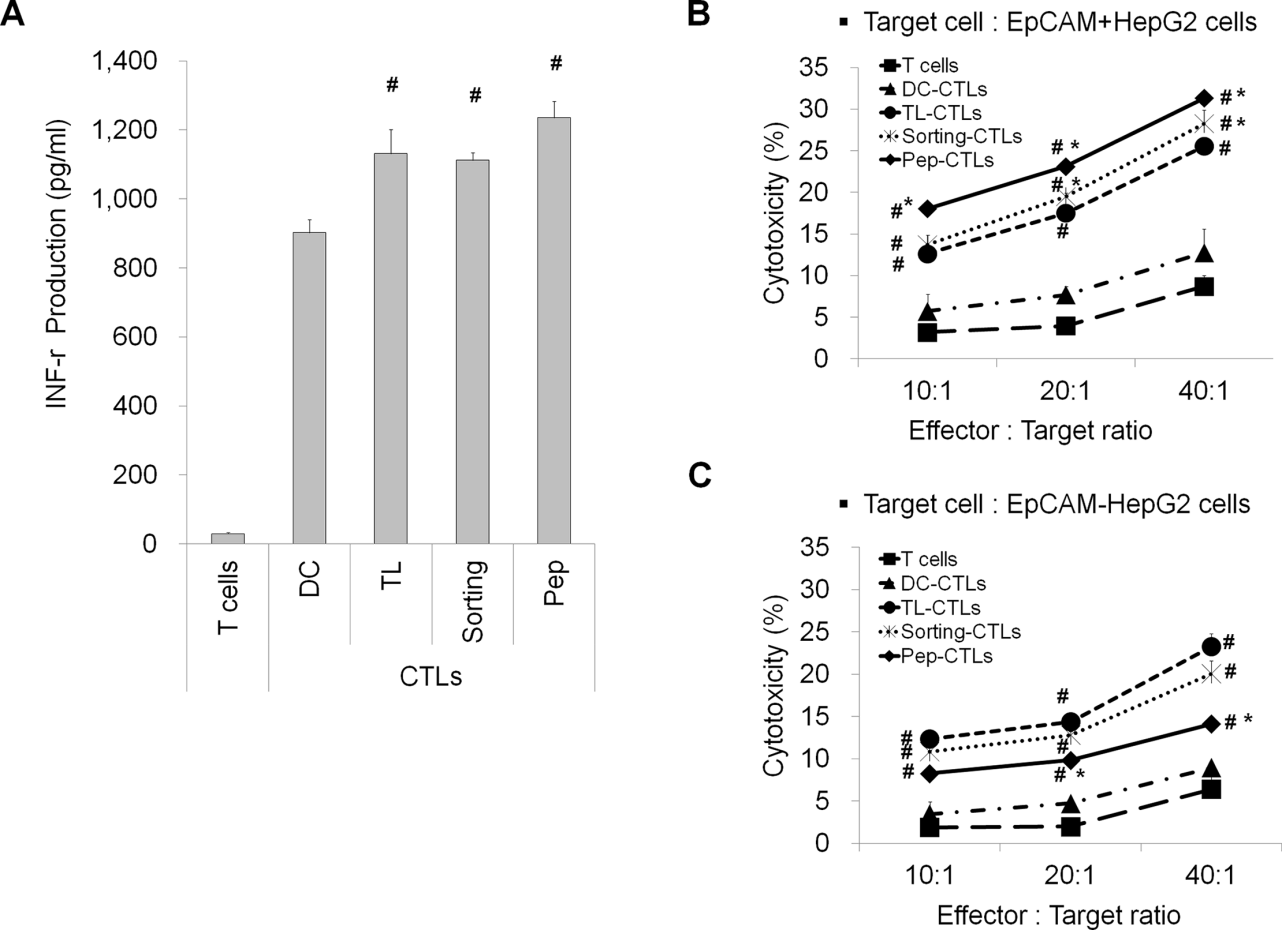
Characterization of the EpCAM peptide-specific CTL responses against HepG2 CSCs. (A) IFN-γ release by antigen-specific CTLs stimulated by antigens. Naïve T cells stimulated by co-culturing with tumor antigen-pulsed DCs were analyzed by ELISA for their production of IFN-γ. (B and C) Results from a cytotoxicity assay of CD8^+^ T cells activated by tumor antigen-pulsed DCs. Different DCs were co-cultured at a ratio of 1:5 with lymphocytes for 5 days. The non-adherent cells were collected and counted as effector cells. The effector cells were co-cultured with the EpCAM positive (B) and EpCAM negative (C) HepG2 cells at different ratios for 6 h at 37°C in a 5% CO_2_ incubator. The data are expressed as the mean±SD, and significant differences between the treated groups were detected using ANOVA followed by the Dunnett’s test. ^#^ *p*<0.05 compared with the DCs or DC-CTLs, and * *p*<0.05 compared with the TL-CTLs.

To confirm the activation of lymphocytes by EpCAM peptide-stimulated DCs, we investigated the levels of IFN-γ secreted by T cells following stimulated with tumor antigens. To generate antigen-specific CTLs from healthy donors, DCs loaded with various antigens were co-cultured with T cells at a DC: T cell ratio of 1:5 for 5 days. T cells stimulated with EpCAM peptide-loaded DCs produced significantly higher levels of IFN-γ than did T cells stimulated with TL- or Sorting-DCs (Fig 4A). The data suggest that DC vaccination using EpCAM peptides can elicit a strong antigen-specific CTL response.

To determine whether the stimulated T cells contained antigen-specific CTLs, we performed cytotoxicity analyses. FACS analysis was used to compare the cytotoxic activities of DC-CTLs, TL-CTL (HepG2 cell lysate stimulated DC-CTLs), Sorting-CTLs (EpCAM^+^ HepG2 cell lysate stimulated DC-CTLs) and Pep-CTLs (EpCAM-1 peptide stimulated DC-CTLs) targeting EpCAM^+^ HepG2 and EpCAM^-^ HepG2 cells. Pep-CTLs had the most efficient cytotoxic activity against EpCAM^+^ HepG2 cells (Fig 4B). However, TL-CTLs were more efficient than Pep-CTLs against EpCAM^-^ HepG2 cells (Fig 4C).

These results indicate that CTLs stimulated by EpCAM peptide-pulsed DCs can recognize the EpCAM present on HepG2 cells in the context of HLA-A2 and kill EpCAM-positive HepG2 tumor cells.

### DC vaccination using EpCAM peptides inhibits HepG2 cell-induced tumor growth

**Fig 5:**
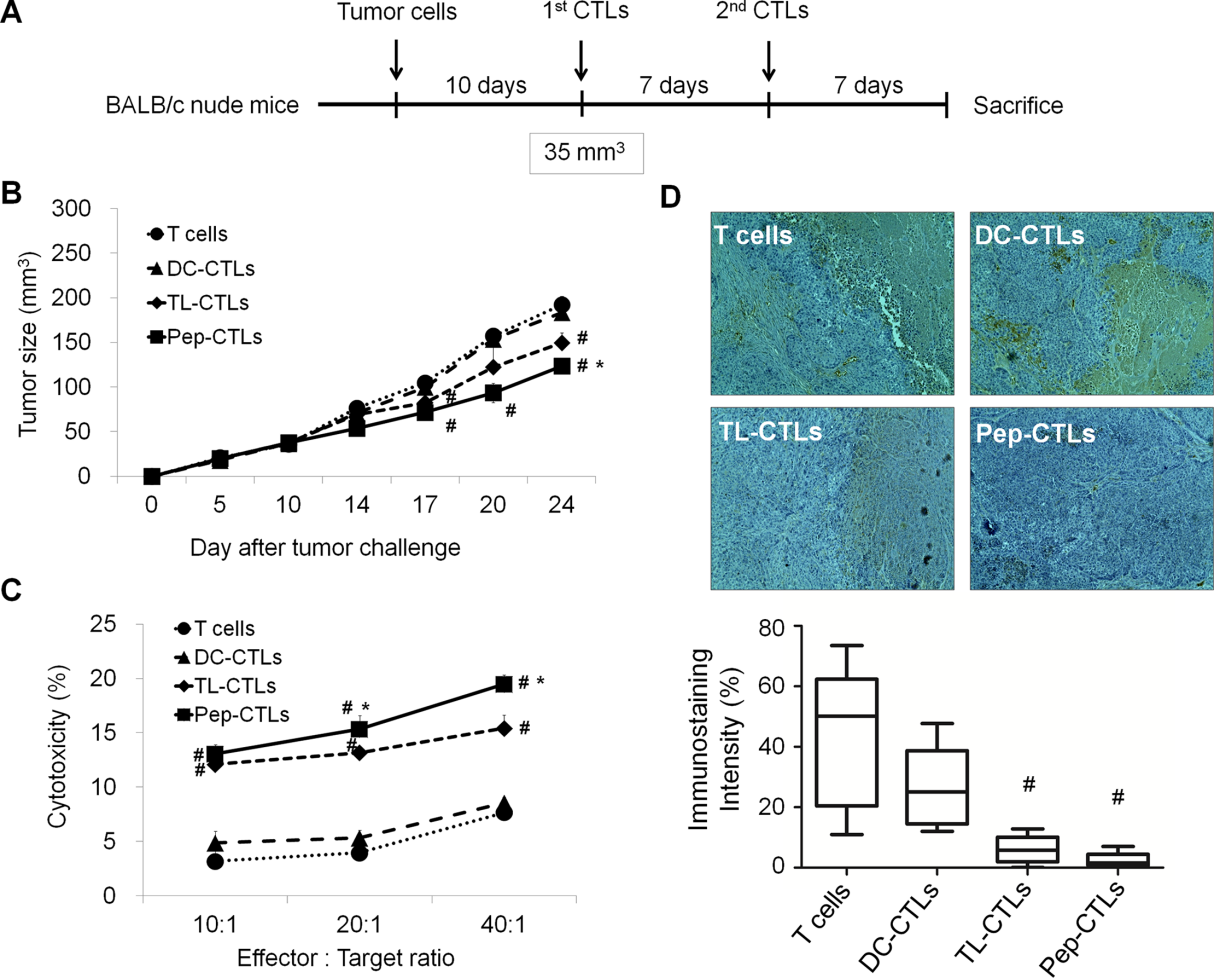
The antitumor efficacy of the EpCAM peptide-pulsed, DC-induced CTLs in HepG2 tumor-bearing mice. (A) The experimental setup and showing the injection schedule for the tumor-associated antigen-pulsed, DC-induced CTLs. (B) Inhibition of tumor growth analysis. Tumor-bearing BALB/c nude mice were treated with intravenous injections of Pep-CTLs (EpCAM peptide stimulated DC-CTLs), TL-CTLs (HepG2 cell lysate stimulated DC-CTLs), DC-CTLs and T cells on days 10 and 17 after tumor implantation. (C) EpCAM^+^ HepG2-specific cytotoxicity. Lymphocytes were isolated from the Pep-CTL-, TL-CTL-, DC-CTL- or T cell-treated mice on day 14 post-inoculation. (D) Immunohistochemistry with anti-EpCAM antibodies in HepG2 tumor tissue. Tumor regions of a section from a tumor isolated on day 24 after tumor implantation. Original magnification, 200×. The data are expressed as the mean±SD, and significant differences between the treated groups were detected using ANOVA followed by the Dunnett’s test. ^#^*P*<0.05 compared with the DC-CTLs, and * *p*<0.05 compared with the TL-CTLs.

To investigate whether EpCAM peptide-pulsed CTLs targeting HepG2 CSCs can induce antitumor immunity in vivo, we established a HepG2 hepatoma cancer model. In the present study, BALB/c nude mice received adoptive immunotherapy by receiving an intravenous injection of Pep-CTLs on days 10 and 17 after tumor implantation. The control groups were injected with TL-CTLs, DC-CTLs or T cells (Fig 5A).

As shown in Fig 5B, the tumors were smallest in the Pep-CTL group at 24 days, followed by the TL-CTL group, the DC-CTL group and the T cell group. To determine whether the relative protective effect of Pep-CTL vaccination was due to tumor-specific immunity, we performed a CTL assay. Tumor-specific CTLs against EpCAM^+^ HepG2 cells were evaluated with by FACS analysis using lymphocytes from the four treated groups. The percentage of EpCAM^+^ HepG2 cells lysed by the lymphocytes from mice treated with Pep-CTLs increased significantly as the ratio of effectors to targets increased (Fig 5C). To examine the effects of Pep-CTLs on EpCAM expression in tumor tissue, we performed immunohistochemical analyses. Tumor tissue from the EpCAM peptide-CTL group showed weak expression, as determined by immunohistochemical staining of EpCAM (Fig 5D).

These results indicate that EpCAM peptide-CTLs inhibit tumor growth in vivo and induce specific immune responses that target CSCs.

## Discussion

DCs are professional APCs that are present in small numbers in all body tissues [25], and they are responsible for capturing and presenting antigens for the initiation of T cell immune responses. Tumor antigens in the form of peptides, proteins, DNA and RNA have been used to sensitize DCs [26]. Recent studies have demonstrated that CSCs are closely related to tumor occurrence, progression, metastasis, recurrence, drug resistance and immune evasion [27–29]. Therefore, in this study, we prepared EpCAM^+^ HepG2 lysates and EpCAM peptides from hepatoma stem-like cells for use in antigen-loaded DC-based vaccines. Human DCs pulsed with EpCAM peptide antigens effectively induced EpCAM peptide-specific CTLs in vitro, and these CTLs effectively killed EpCAM-expressing HepG2 cells.

In general, because the recognition of antigens presented by DCs by TCR of T lymphocytes is limited by MHC molecules, the killing of target cells by activated CTLs is also limited by MHC molecules [30]. The affinity with which an epitope binds to an MHC molecule plays a key role in determining its immunogenicity [31], and high affinity MHC epitope interactions tend to be associated with higher immune responsiveness. As shown in Table 1, we selected EpCAM peptides with high affinity to HLA-A2 molecules and generated EpCAM peptide-specific CTLs using EpCAM peptide-pulsed DCs as APCs. We found that EpCAM^+^ HepG2 cells express high levels of HLA-ABC (MHC-I) and HLA-DR (MHC-II) molecules. Additionally, EpCAM peptides had a stronger binding affinity for the HLA-A2 molecule than did the EpCAM^+^ HepG2 cells in a T2 cell binding assay, suggesting that EpCAM peptides are more immunogenic (Fig 2).

Tumor-specific CD8-positive CTLs are the most important effector cells for antitumor responses [32, 33]. In the present study, human mDCs from a healthy HLA-A2+ donor were loaded with various antigens (HepG2 cell lysates, EpCAM^+^ HepG2 cell lysates and EpCAM peptides) (Fig 3) and co-cultured with T cells to generate antigen-specific CTLs. The results showed that the EpCAM peptide antigens induced higher levels of T cell IFN-γ secretion and that the EpCAM peptide CTLs had the most efficient cytotoxic activity against EpCAM^+^ HepG2 cells. These results indicate that CTLs stimulated by EpCAM peptide-pulsed DCs can recognize the EpCAM presented by HepG2 cells in the context of HLA-A2and kill EpCAM positive HepG2 tumor cells (Fig 4). Moreover, in MCF-7 human breast adenocarcinoma cells, the CD44-1 peptide had the strongest binding affinity for the HLA-A2 molecule, and CD44-1 peptide stimulated DC-CTLs exhibited the most efficient cytotoxic activity against CD44^+^ MCF-7 cells (Fig 1C).

Finally, we investigated whether the EpCAM peptide-pulsed, DC-induced CTLs targeting CSCs could induce antitumor immunity in vivo. The results showed that vaccination of nude mice with EpCAM peptide-CTLs delayed tumor growth induced by HepG2 cells. The FACS-based cytotoxicity assay of tumor-specific CTLs against EpCAM^+^ HepG2 cells using lymphocytes from the four treatment groups showed that EpCAM peptide-CTLs increased the cytotoxic activity significantly as the ratio of effectors to targets increased. The tumor tissue from the EpCAM peptide-CTLs group showed weak expression, as determined by EpCAM immunohistochemical staining. These results indicate that vaccination with CSC peptide-primed DCs can induce specific immune responses that target CSCs in the HepG2 hepatoma tumor model (Fig 5).

Our present study demonstrated that CSC peptides are present on HLA-A2 molecules and that the killing of CSCs expressing human breast and HCC CSCs by peptide-specific CTLs is restricted by HLA-A2. Therefore, CSC peptides may be better sources of antigens for cancer immunization that are the sources that are currently used. Additionally, CSC peptide-pulsed, DC-induced CTLs can be used for vaccination immunotherapy to eliminate human breast and HCC CSCs.

## Supporting information

S1 File ARRIVE checklist. (DOCX)
