# Supplementary material for: EpCAM peptide-primed dendritic cell vaccination confers significant anti-tumor immunity in hepatocellular carcinoma cells
Source: PLoS One. 2018 Jan 3;13(1):e0190638. doi: 10.1371/journal.pone.0190638 (PMC5752035; doi:10.1371/journal.pone.0190638)

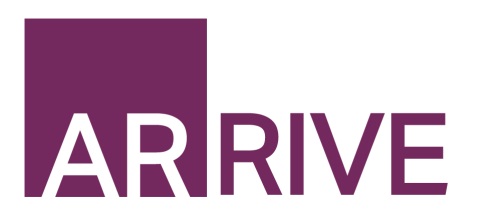


The ARRIVE Guidelines Checklist

Animal Research: Reporting In Vivo Experiments

Carol Kilkenny^1^, William J Browne^2^, Innes C Cuthill^3^, Michael Emerson^4^ and Douglas G Altman^5^

*^1^The National Centre for the Replacement, Refinement and Reduction of Animals in Research, London, UK, ^2^School of Veterinary Science, University of Bristol, Bristol, UK, ^3^School of Biological Sciences, University of Bristol, Bristol, UK, ^4^National Heart and Lung Institute, Imperial College London, UK, ^5^Centre for Statistics in Medicine, University of Oxford, Oxford, UK.*

|  | | ITEM | RECOMMENDATION | Section/ Paragraph |
| --- | --- | --- | --- | --- |
| 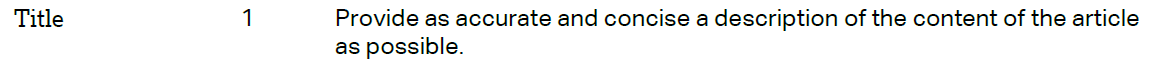 | | | Title |  |
| 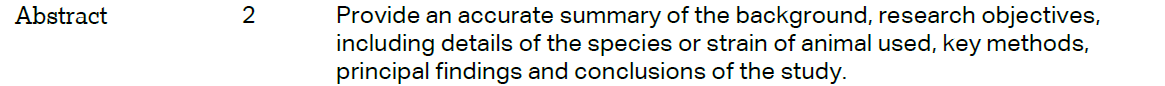 | | | Abstract |  |
| INTRODUCTION | | |  |  |
| 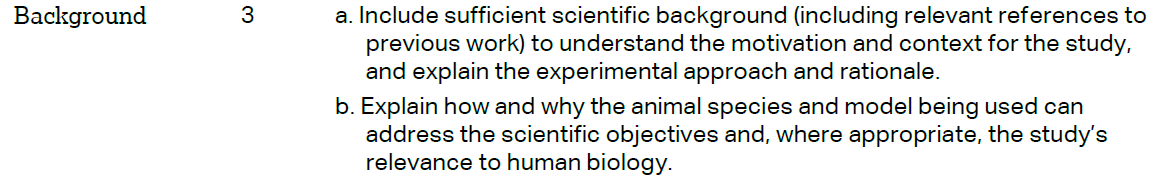 | | | Paragraphs 1-3  Paragraph 3 |  |
| 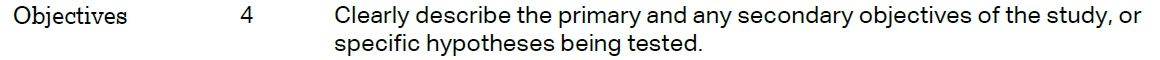 | | | Paragraph 4 |  |
| METHODS | | |  |  |
| 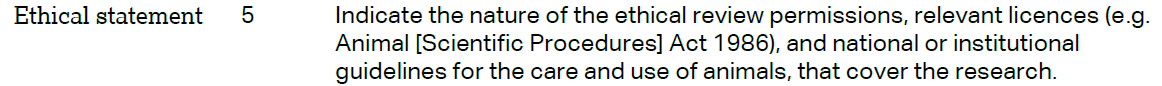 | | | Subhead 11 |  |
| 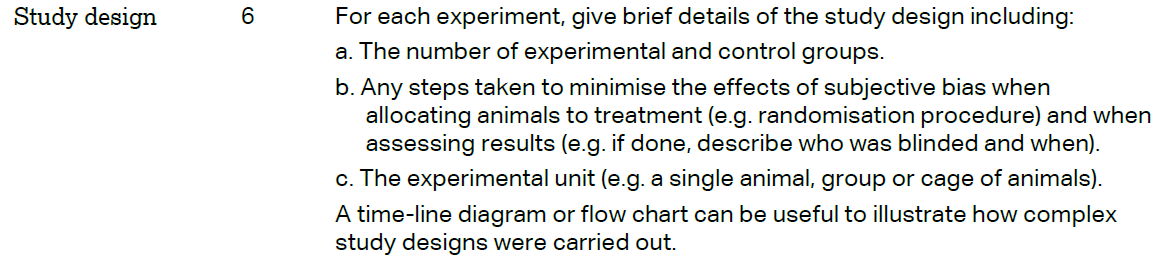 | | | Subhead 11  Figure 5 |  |
| 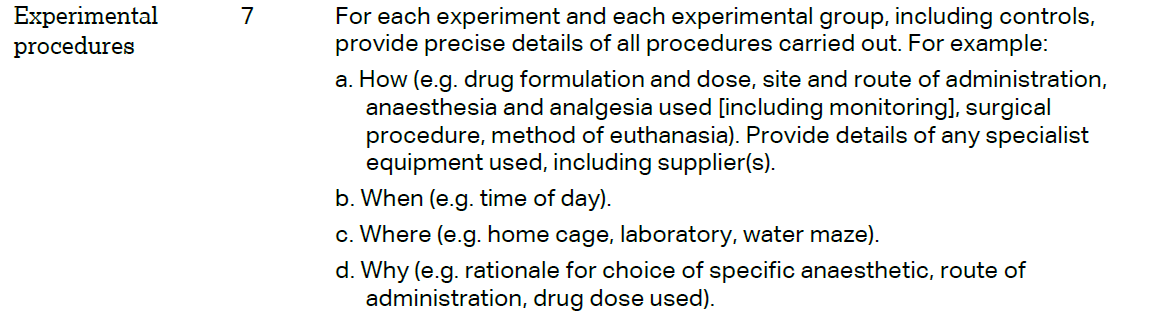 | | | Subhead 11  Figure 5 |  |
| 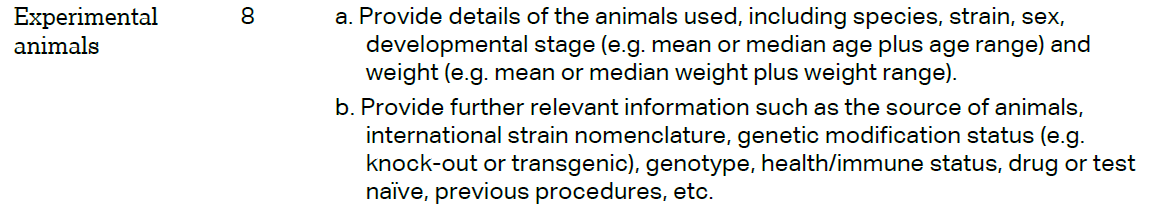 | | | Subhead 11 |  |

The ARRIVE guidelines. Originally published in *PLoS Biology*, June 2010^1^

| 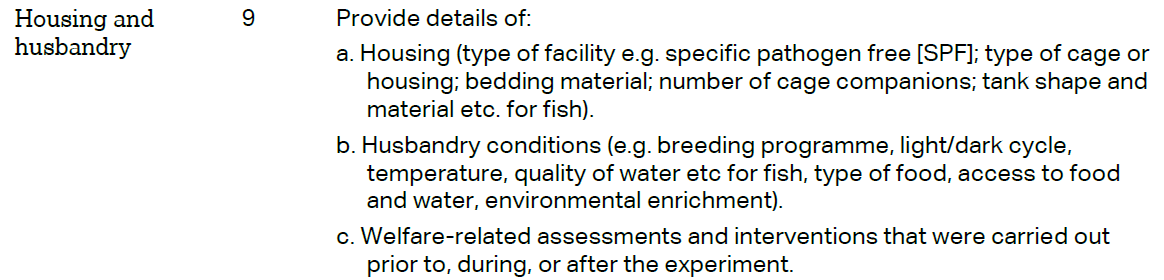 | Subhead 11 | |
| --- | --- | --- |
| 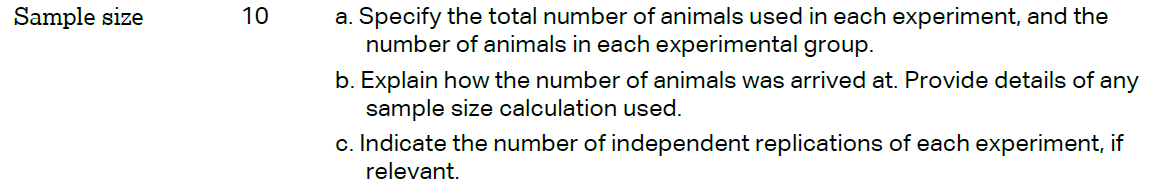 | Subhead 11 | |
| 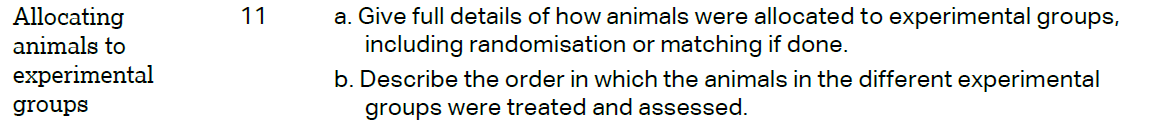 | Subhead 11  Figure 5 | |
| 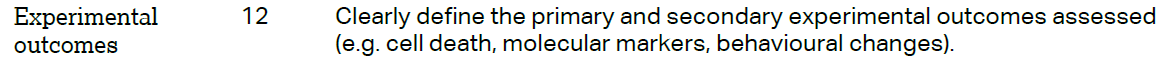 |  | |
| 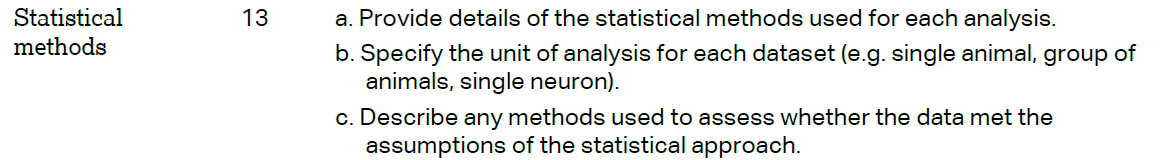 | Subhead 13 | |
| RESULTS |  | |
| 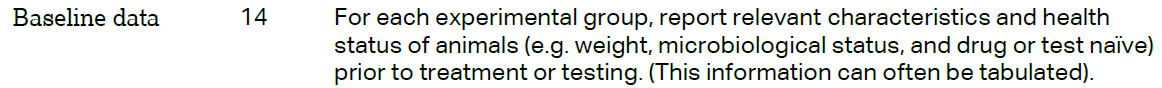 | Subhead 6 | |
| 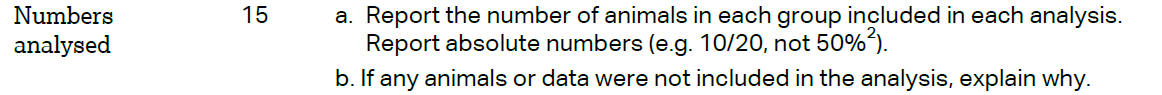 | Methods  Subhead 11 | |
| 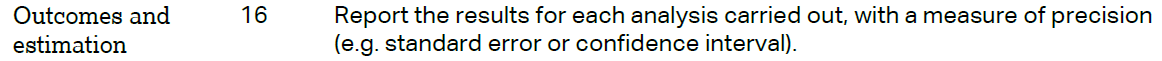 | Subhead 6  Figure 5 | |
| 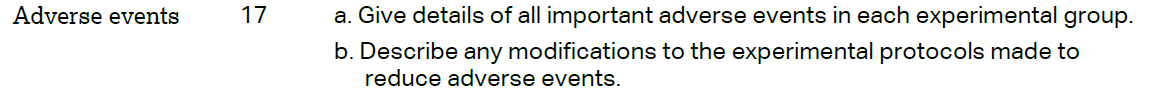 |  | |
| DISCUSSION |  | |
| 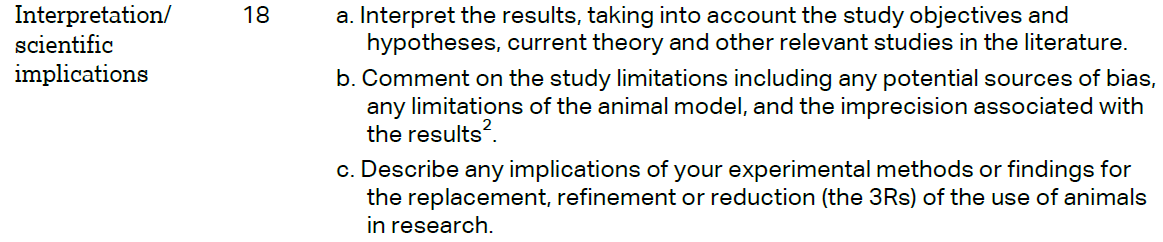 | Throughout | |
| 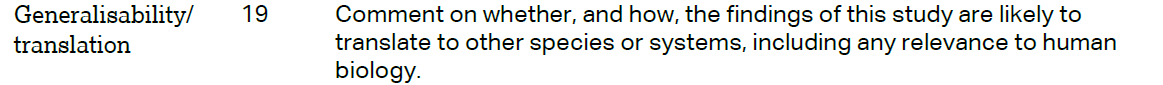 | Paragraph 4 | |
| 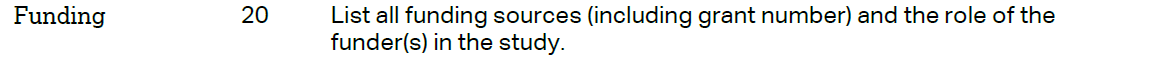 | | Acknowledgement |


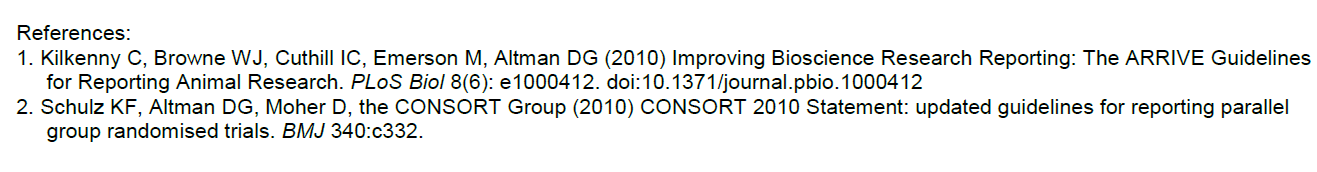

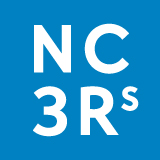

Supplement: S1 File — (DOCX) [file pone.0190638.s001.docx]
